# Thoracic ultrasound combined with low-dose computed tomography may represent useful screening strategy in highly exposed population in the industrial city of Taranto (Italy)

**DOI:** 10.3389/fmed.2023.1146807

**Published:** 2023-05-16

**Authors:** Carla Maria Irene Quarato, Elisa Dama, Michele Maggi, Beatrice Feragalli, Cristina Borelli, Anna Del Colle, Marco Taurchini, Evaristo Maiello, Salvatore De Cosmo, Donato Lacedonia, Maria Pia Foschino Barbaro, Giovanna Elisiana Carpagnano, Giulia Scioscia, Paolo Graziano, Rosalinda Termine, Elisabettamaria Frongillo, Sonia Santamaria, Mariapia Venuti, Maria Arcangela Grimaldi, Stefano Notarangelo, Annarita Saponara, Massimiliano Copetti, Tommaso Colangelo, Roberto Cuttano, Dimitrios Macrodimitris, Francesco Mazzarelli, Michela Talia, Antonio Mirijello, Luca Pazienza, Rita Perna, Anna Simeone, Doriana Vergara, Antonio Varriale, Massimo Carella, Fabrizio Bianchi, Marco Sperandeo

**Affiliations:** ^1^Department of Medical and Surgical Sciences, Institute of Respiratory Diseases, Policlinico Universitario “Riuniti” di Foggia, University of Foggia, Foggia, Italy; ^2^Cancer Biomarkers Unit, Institute for Stem-Cell Biology, Regenerative Medicine and Innovative Therapies (ISBReMIT), IRCCS Fondazione Casa Sollievo della Sofferenza, San Giovanni Rotondo, Foggia, Italy; ^3^Department of Emergency Medicine and Critical Care, Emergency Medicine Unit, IRCCS Fondazione Casa Sollievo della Sofferenza, San Giovanni Rotondo, Foggia, Italy; ^4^Department of Medical, Oral and Biotechnological Sciences, Radiology Unit, “G. D’Annunzio,” University of Chieti-Pescara, Chieti, Italy; ^5^Department of Radiology, IRCCS Fondazione Casa Sollievo della Sofferenza, San Giovanni Rotondo, Foggia, Italy; ^6^Unit of Thoracic Surgery, IRCCS Fondazione Casa Sollievo della Sofferenza, San Giovanni Rotondo, Foggia, Italy; ^7^Unit of Oncology, IRCCS Fondazione Casa Sollievo della Sofferenza, San Giovanni Rotondo, Foggia, Italy; ^8^Department of Internal of Medicine, IRCCS Fondazione Casa Sollievo della Sofferenza, San Giovanni Rotondo, Foggia, Italy; ^9^Department of Basic Medical Sciences, Neuroscience and Sense Organs, Section of Respiratory Disease, University “Aldo Moro” of Bari, Bari, Italy; ^10^Unit of Patology, IRCCS Fondazione Casa Sollievo della Sofferenza, San Giovanni Rotondo, Foggia, Italy; ^11^Unità Sanitaria Locale (ASL) di Potenza, Potenza, Italy; ^12^Unit of Biostatistics, IRCCS Fondazione Casa Sollievo della Sofferenza, San Giovanni Rotondo, Foggia, Italy; ^13^Internal Medicine, “San Pio” Hospital, Azienda Sanitaria Locale (ASL) di Castellaneta, Castellaneta, Italy; ^14^Internal Medicine, “Bernardini” Nursing Home, Taranto, Italy; ^15^Clinical Trial Office—Scientific Direction, IRCCS Fondazione Casa Sollievo della Sofferenza, Foggia, Italy; ^16^Division of Medical Genetics, IRCCS Fondazione Casa Sollievo della Sofferenza, San Giovanni Rotondo, Foggia, Italy; ^17^Unit of Interventional and Diagnostic Ultrasound of Internal Medicine, IRCCS Fondazione Casa Sollievo della Sofferenza, San Giovanni Rotondo, Foggia, Italy

**Keywords:** transthoracic ultrasound, high-resolution computed tomography, lung cancer, interstitial lung abnormalities, screening protocol, environmental exposure

## Abstract

**Objectives:**

We validated a screening protocol in which thoracic ultrasound (TUS) acts as a first-line complementary imaging technique in selecting patients which may deserve a second-line low-dose high resolution computed tomography (HRCT) scan among a population of asymptomatic high-risk subjects for interstitial lung abnormalities (ILA) and lung cancer. Due to heavy environmental pollution burden, the district Tamburi of Taranto has been chosen as “case study” for this purpose.

**Methods:**

From July 2018 to October 2020, 677 patients aged between 45 and 65 year and who had been living in the Tamburi district of Taranto for at least 10 years were included in the study. After demographic, clinical and risk factor exposition data were collected, each participant underwent a complete TUS examination. These subjects were then asked to know if they agreed to perform a second-level examination by low-dose HRCT scan.

**Results:**

On a total of 167 subjects (24.7%) who agreed to undergo a second-level HRCT, 85 patients (50.9%) actually showed pleuro-pulmonary abnormalities. Interstitial abnormalities were detected in a total of 36 patients on HRCT scan. In particular, 34 participants presented subpleural ILAs, that were classified in the fibrotic subtype in 7 cases. The remaining 2 patients showed non-subpleural interstitial abnormalities. Subpleural nodules were observed in 46 patients. TUS showed an overall diagnostic accuracy of 88.6% in detecting pleuro-pulmonary abnormalities in comparison with HRCT scan, with a sensitivity of 95.3%, a specificity of 81.7%, a positive predictive value of 84.4% and a negative predictive value of 94.4%. The matched evaluation of specific pulmonary abnormalities on HRTC scan (i.e., interstitial abnormalities or pulmonary nodules) with determinate sonographic findings revealed a reduction in both TUS sensibility and specificity. Focusing TUS evaluation on the assessment of interstitial abnormalities, a thickened pleural line showed a sensitivity of 63.9% and a specificity of 69.5%, hypoechoic striae showed a sensitivity of 38.9% and a specificity of 90.1% and subpleural nodules showed a sensitivity of 58.3% and a specificity of 77.1%. Regarding to the assessment of subpleural nodules, TUS showed a sensitivity of 60.9% and a specificity of 81.0%. However, the combined employment of TUS examination and HRCT scans allowed to identify 34 patients with early subpleural ILA and to detect three suspicious pulmonary nodules (of which two were intraparenchymal and one was a large subpleural mass), which revealed to be lung cancers on further investigations.

**Conclusion:**

A first-line TUS examination might aid the identification of subjects highly exposed to environmental pollution, who could benefit of a second-line low-dose HRCT scan to find early interstitial lung diseases as well as lung cancer.

**Protocol registration code:**

PLEURO-SCREENING-V1.0_15 Feb, 17.

## Introduction

The city of Taranto, in the southeast of the Italian peninsula, is historically considered the industrial capital of the Apulia region and the Mediterranean. The intense industrial activity has earned this city the inclusion in the list of the so called “sites of national interest” (SNI), which requires special monitoring and reclamation programs for the great risk of environmental crisis (1). Several researches have assessed a heavy environmental diffusion of contaminants in the industrial area of Taranto, among which, particulate matter (PM_2.5_ and PM_10_), nitric dioxide (NO_2_), sulfur dioxide (SO_2_), carbon monoxide (CO), polycyclic aromatic hydrocarbons (PAH), benzo(a)pyrene (BaP), benzodioxins (BDO) and polychlorinated dibenzodioxins (PCDDs) (2–6). The various reports of the epidemiological study SENTIERI confirmed particular excesses of incidence and mortality for lung cancer, mesothelioma of the pleura and respiratory diseases in the SNI of Taranto (7–10). The cohort study of Mataloni et al. (11). revealed a greater increase in the risk of mortality and hospitalizations for cardiovascular and respiratory diseases among the residents of two neighborhoods closest to the steel plant (i.e., Tamburi and Borgo).

Screening strategies using low-dose high resolution computed tomography (HRCT) have been shown to be able to reduce mortality in subjects at high risk for lung cancer and respiratory diseases (12–17). However, as the cost–benefit ratio resulting from this practice is still unclear, an annual lung cancer screening employing low-dose HRCT in high-risk patients is not yet recommended and, therefore, not reimbursed by the National Health System of most part of the European countries (18). Lung cancer screening through periodic chest X-ray and/or sputum cytology has not shown any benefit (18).

Thoracic ultrasound (TUS) is widely accessible and less expensive than other imaging methods. In addition, TUS does not expose patients to ionizing radiation, which is a relevant characteristic when used for screening purposes. The normal lung is scarcely penetrable by ultrasounds due to its air content (19). However, a variety of peripheral pleuro-pulmonary abnormalities become accessible to TUS when they are strictly adherent to or directly involve the parietal pleural surface (19, 20). To this regard, current evidence suggest a promising role for TUS in the detection of peripheral lung consolidations (i.e., pneumonia and neoplasm) and in the early screening of interstitial lung diseases (21–23). Tierney et al. (24) have shown that TUS can even outperform portable chest X-ray in the detection of pleuro-pulmonary abnormalities.

On this background, our goal was to validate a screening protocol that includes the use of TUS in a population highly exposed to environmental pollutants, to identify subjects who could benefits of a second-line HRCT scan for the early detection of interstitial lung disease and lung cancer. We designed a pilot study in the district Tamburi of Taranto, with the aim of validating this strategy. HRCT scan was considered as the “gold standard” test against which to assess TUS accuracy.

## Materials and methods

### Design and methods of the screening protocol

This study was approved by the Research Ethics Committee of the Istituto di Ricovero e Cura a Carattere Scientifico (IRCCS) “Fondazione Casa Sollievo della Sofferenza” in San Giovanni Rotondo, Italy (Prot. N 66/CE IRCCS CSS: PLEURO-SCREENING-V1.0–15 Feb, 2017), and it is still ongoing.

The inclusion criteria are: (1) having spent a period of at least 10 years residing in the neighborhood Tamburi of Taranto; (2) an age comprised within 45–65 year; and (3) agreeing to participate in the study by giving written informed consent.

The exclusion criteria are: (1) being unable to undergo HRCT scans; (2) being pregnant; (3) having respiratory symptoms or known acute respiratory diseases (i.e., bronchitis, pneumonia); (4) presenting pulmonary fibrotic sequelae (i.e., history of previous tuberculosis or radiation therapy); (5) having a known history of interstitial lung disease; (6) having had direct contact with known COVID-19 cases or have been affected by COVID-19 pneumonia.

Subjects who meet the inclusion criteria are enrolled at the “Divino Lavoratore” Parish in the Tamburi district of Taranto, where a special medical clinic was authorized and set up following all the rules provided by the national health system. The project is completely no-profit.

At the initial visit, demographic, clinical and risk factor exposition data are collected. During the same visit, each participant undergoes TUS examination. A Flow/Volume spirometry is also performed for completeness.

Once the preliminary visit is over, the enrolled subjects are contacted to ask them if they agree to be scheduled for a second-level examination by HRCT.

### Pulmonary function tests

Pulmonary function tests (PFTs) are performed using a portable spirometer (Viasys Healthcare Microlab 3,500 Spirometer, Camarillo, CA, USA). The obtained forced expiratory volume in the first second (FEV_1_) and forced vital capacity (FVC), expressed as a percentage of predicted, consisted in the best of three reproducible measurements of maximally forced inspiratory and expiratory maneuvers. The presence of obstruction is diagnosed on the basis of a FEV_1_/FVC ratio less than 70%. The possibility of having a restrictive ventilatory defect is suggested by both FEV_1_ and FVC values less than 80% of predicted (25).

### Thoracic ultrasound examination

For TUS examination is used a Mindray-7 (Mindray Medical Italy S.R.L, Trezzano sul Naviglio, Milan, Italy) or, alternatively, an Esaote MyLab-30 (Esaote, Genoa, Italy) ultrasound system. Each TUS examination is performed with a low multi-frequency convex probe (3.5–5 MHz), properly setting the ultrasound scanner for the study of the adult thorax, as we previously reported elsewhere (22, 26). Technical parameters employed are: time gain compensation (TGC) adjustment no more than 55%, electronic focus of the beam placed in correspondence of the pleural line and activation of the tissue harmonic imaging. Patients’ chest is examined from bottom (i.e., starting from the identification of the costo-diaphragmatic sinuses) to top, with longitudinal and transverse intercostal scans, along the longitudinal anatomical lines of demarcation (i.e., paravertebral and hemiscapular lines, posteriorly; postero-axillary, mid-axillary and anterior-axillary lines, laterally; hemi-clavicular and parasternal line, anteriorly), in a sitting position (19, 22). TUS examination, conducted in double blind by two sonographers (with 10 and 30 years of experience in TUS, respectively) is considered positive when it shows thickening of the hyperechoic pleural line, subpleural nodules and hypoechoic striae. The hyperechoic pleural line is judged as “thickened” when it measures more than 3 mm (28). Subpleural nodules are defined as subpleural hypo-echoeic lesions, round or oval in shape. Hypoechoic striae are defined as hypo-echoeic lesions extending more in width than in height (22). Although B-lines or ring-downs (continuous and parallel hyperechoic stripes, arising from the pleural line and extending indefinitely on the screen) have been described as an ultrasound sign of pulmonary fibrosis, they were not valued in this study. Indeed, an unspecific increase in B-lines may be found in several other pathological conditions [e.g., pulmonary congestion, pulmonary contusion, pneumonia, acute respiratory distress syndrome, acute exacerbation of chronic obstructive pulmonary disease and asthma and neoplastic lymphangitis (27)] and even in healthy individuals [generally at the bases, where the hydrostatic pressure gives a more fluid-rich interstitium (28)]. Furthermore, the B-lines’ “count” displays an excessive inter- and intra-observers variability and cannot be considered a scientifically useful procedure (29, 30).

### Chest high resolution computed tomography examination

Subjects in agreement to perform a second level HRCT scan are referred at the Radiology Unit of the IRCCS Fondazione Casa Sollievo della Sofferenza hospital in San Giovanni Rotondo, Italy. The exams are performed and interpreted by a dedicated radiologist with at least 30 years of experience in lung imaging, using a 64-channel multi-detector CT scanner (Toshiba, Tokyo, Japan). The detailed parameters for CT acquisition are: voltage, 120 kVp; standard amperage (reference mAs, 60–120); thickness, 0.5 mm; reconstruction interval, 0.5–1.0 mm. All CT images are acquired on complete inspiration, with the patient in a supine position and without contrast. HRCT scans evaluation is specifically focused on the detection of pulmonary nodules and interstitial abnormalities. The examination is completed by the assessment of eventual pleural changes, including circumscribed pleural thickening, pleural plaques and pleural effusion. According to the Fleischner Society (31), incidentally detected interstitial lung abnormalities (ILAs) are defined as non-subpleural and non-fibrotic (i.e., ground-glass opacity and reticular opacities without a predominant subpleural localisation), subpleural non-fibrotic (i.e., ground-glass opacity and reticular opacities with a predominant subpleural localisation without evidence of fibrosis) and subpleural fibrotic (i.e., ground-glass opacity and reticular opacities with a predominant subpleural localisation and with evidence of fibrosis, including traction bronchiectasis, architectural distortion, and honeycombing). Centrilobular nodules and bronchiolocentric interstitial ground-glass opacifications were excluded from the list of ILAs features as more likely related to smoking habits (32, 33).

The dedicated radiologist is blinded to corresponding TUS examination results while interpreting HRCT scan findings in each patients.

### Statistic analysis

Data collected are presented as number (*n*) and percentage (%) for categorical variables, and as median with first and third quartiles (Q1;Q3) for continuous variables. Statistical differences in terms of baseline and exposure characteristics for the subsets of patients with TUS negative/positive and HRCT performed/not performed were evaluated using the Fisher’s exact test and the Wilcoxon test for categorical and continuous variables, respectively. Differences in proportions of TUS findings were evaluated through a *Z*-test for proportions. A value of *p* less than 0.05 was considered statistically significant. TUS sensitivity, specificity, positive and negative predicted values, and likelihood ratios for positive and negative test were calculated with a 95% confidence interval (CI), considering HRCT as “the gold standard test.” Cohen’s kappa coefficient was calculated to determine inter-reader agreement in the interpretation of TUS findings. All statistical analyses were performed using SAS software, version 9.4 (SAS Institute, Inc., Cary, NC).

## Results

### Study flow of participants

**Figure 1:**
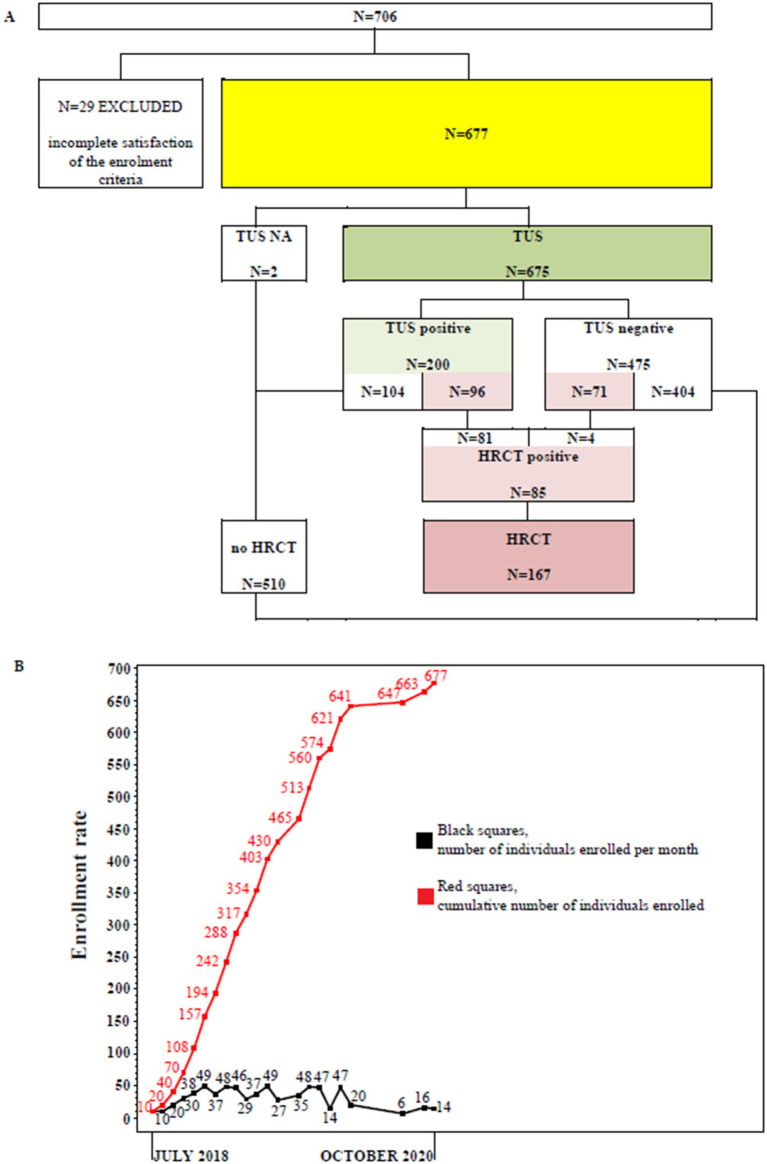
**(A)** Study flow chart. TUS, transthoracic ultrasound examination; HRCT, chest high resolution computed tomography examination. **(B)** Enrollment rate between July, 2018 and October, 2020. Black squares represent the number of individuals enrolled per month. Red squares represent the cumulative number of individuals enrolled.

From July 2018 to October 2020 a total of 706 subjects were recruited. Twenty nine participants did not meet the study protocol inclusion criteria and were excluded. Finally, 677 subjects (291 men, 386 women; median age: 55 years) were included in the study, of whom 675 underwent TUS examination. A total of 167 subjects (24.7%) have agreed to undergo both a TUS examination and a second-level HRCT scan. Eighty five out of 167 (50.9%) individuals showed a positive second-level HRCT result. The flow of participants through the study is detailed in Figure 1A. The enrollment rate from July 2018 to October 2020 is shown in Figure 1B.

**Figure 2:**
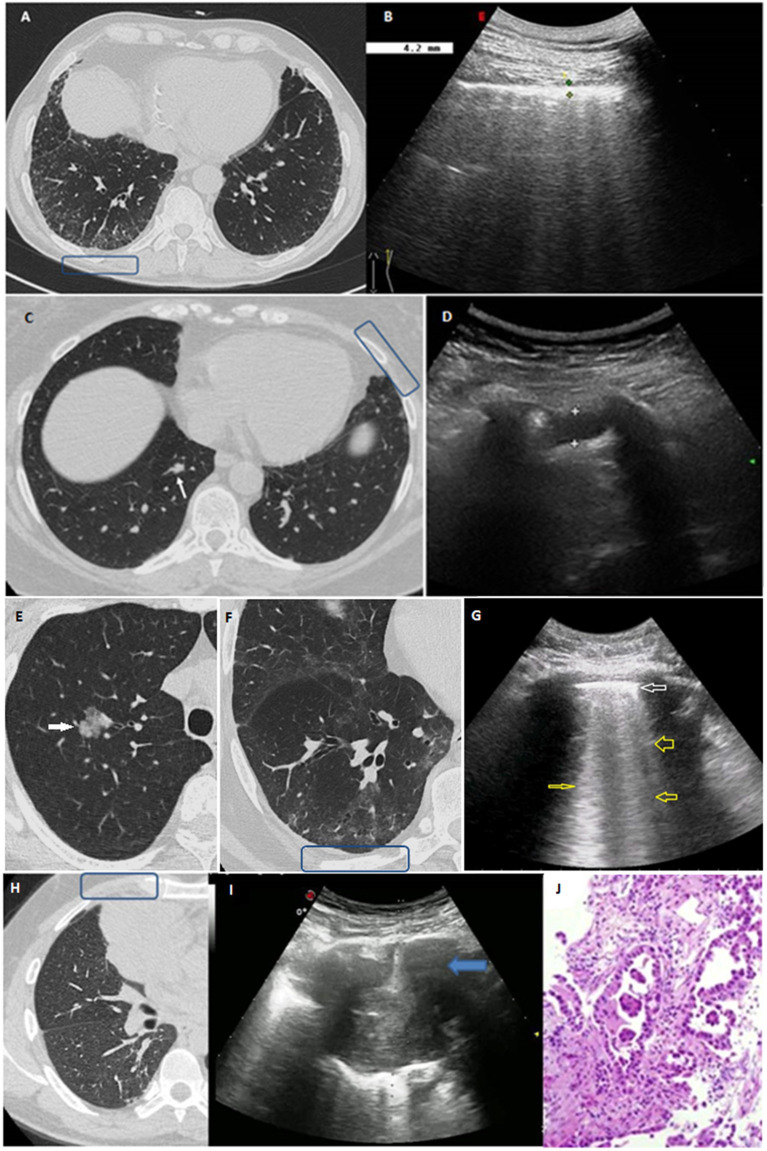
**(A,B)** Clinical case 1: **(A)** HRCT scan displaying bilateral GGOs and superimposed reticular opacities in the subpleural regions of both lower lobes, especially on the right (blue box). **(B)** The corresponding TUS examination at the level of right posterior para-vertebral lower scan showed a thickened hyperechoic pleural line. **(C,D)** Clinical case 2: **(C)** Axial HRCT scan showing a subtle focal pleural thickening in the left lung (blue box) and an intraparechymal irregular solid nodule in the right lower lobe (white arrow). **(D)** TUS examination at the level of left anterior hemi-clavicular middle scan showed a hypoechoic stria. **(E–G)** Clinical case 3: **(E)** Axial HRCT scan showing the presence of a 21 mm intraparenchymal part-solid nodule in the right upper pulmonary lobe (white arrow) very suspicious for lung cancer. **(F)** Axial HRCT scan of the right lower lobe showing a subpleural area of GGO with subtle thickening of the intralobular septa and small traction bronchiolectasis indicative of parenchymal distortion (blue box). **(G)** TUS examination was not able to identify the nodule, but TUS scan at the level of right posterior para-vertebral lower zone assessed an irregular thickened hyperechoic pleural line (white arrow) with B line artifacts below (yellow arrows). **(H–J)** Clinical case 4: **(H)** HRCT scan showing the presence of a right lung mass in the middle lobe (blue box). **(I)** Ongoing TUS-guided biopsy at the level of the right anterior hemi-clavicular middle zone showed a lung consolidation. **(J)** The histological diagnosis was invasive lung adenocarcinoma.

### Demographic and clinical characteristics of participants

Demographic and clinical characteristics of the enrolled participants (*N* = 677), together with their exposure to chemical risk factors for pleuro-pulmonary disease, are shown in Table 1. Data are presented for the whole cohort and for the subsets of patients with TUS negative/positive and HRCT performed/not performed.

**Table 1:** Association analysis of cohort subsets [transthoracic ultrasound examination (TUS) negative/positive; chest high resolution computed tomography examination (HRCT) performed/not performed] and demographic, clinical characteristics and exposures to chemical risk factors in the PLEUROSCREENING cohort (*N* = 677).

	Whole cohort *N* = 677	TUS negative *N* = 475	TUS positive *N* = 200	No HRCT *N* = 510	HRCT *N* = 167	Value of *p*^a^	
						TUS positive vs. negative	HRCT vs. no HRCT
Age [years] median (Q1;Q3)	55 (50;60)	55 (50;60)	57 (51;60)	55 (50;60)	57 (50;61)	0.09	0.09
BMI
Underweight	3 (0.4%)	2 (0.4%)	1 (0.5%)	1 (0.2%)	2 (1.2%)	0.60	0.36
Healthy	200 (29.5%)	133 (28.0%)	66 (33.0%)	153 (30.0%)	47 (28.1%)		
Overweight	294 (43.4%)	213 (44.8%)	80 (40.0%)	225 (44.1%)	69 (41.3%)		
Obesity	173 (25.6%)	121 (25.5%)	52 (26.0%)	125 (24.5%)	48 (28.7%)		
NA	7 (1.0%)	6 (1.3%)	1 (0.5%)	6 (1.2%)	1 (0.6%)		
Gender
Male	291 (43.0%)	184 (38.7%)	106 (53.0%)	195 (38.2%)	96 (57.5%)	0.0009	<0.0001
Female	386 (57.0%)	291 (61.3%)	94 (47.0%)	315 (61.8%)	71 (42.5%)		
Respiratory comorbities	70 (10.3%)	49 (10.3%)	19 (9.5%)	47 (9.2%)	23 (13.8%)	0.89	0.11
Cardiovascular comorbities	133 (19.6%)	95 (20.0%)	36 (18.0%)	97 (19.0%)	36 (21.6%)	0.71	0.19
Previous tumor	47 (6.9%)	31 (6.5%)	16 (8.0%)	35 (6.9%)	12 (7.2%)	0.51	0.86
Benign	15 (2.2%)	11 (2.3%)	4 (2.0%)	9 (1.8%)	6 (3.6%)	0.66	0.49
Malignant	31 (4.6%)	19 (4.0%)	12 (6.0%)	25 (4.9%)	6 (3.6%)		
histology NA	1 (0.1%)	1 (0.2%)	-	1 (0.2%)	-		
Screening (last 5 years)
Pap-test (females)	325 (84.2%)	240 (82.5%)	84 (89.4%)	262 (83.2%)	63 (88.7%)	0.14	0.28
Breast (females)	327 (84.7%)	240 (82.5%)	86 (91.5%)	261 (82.9%)	66 (93.0%)	0.0469	0.0429
Colon	122 (18.0%)	80 (16.8%)	42 (21.0%)	83 (16.3%)	39 (23.4%)	0.23	0.0482
Fecal occult blood	80 (11.8%)	52 (10.9%)	28 (14.0%)	56 (11.0%)	24 (14.4%)	0.30	0.27
Urology visit	100 (14.8%)	54 (11.4%)	46 (23.0%)	67 (13.1%)	33 (19.8%)	0.0002	0.0440
PSA (males)	201 (69.1%)	126 (68.5%)	75 (70.8%)	133 (68.2%)	68 (70.8%)	0.79	0.69
Cardiac	444 (65.6%)	306 (64.4%)	136 (68.0%)	329 (64.5%)	115 (68.9%)	0.43	0.35
Skin	129 (19.1%)	94 (19.8%)	35 (17.5%)	99 (19.4%)	30 (18.0%)	0.52	0.73
Spirometry
Restrictive impairment (FEV1 and FVC <80%)	47 (6.9%)	33 (6.9%)	14 (7.0%)	32 (6.3%)	15 (9.0%)	0.65	0.66
Obstructive impairment (FEV1/FVC ratio < 70%)	1 (0.1%)	1 (0.2%)	-	1 (0.2%)	-		
Restrictive-obstructive impairment	3 (0.4%)	1 (0.2%)	2 (1.0%)	3 (0.6%)	-		
Normal	601 (88.8%)	424 (89.3%)	176 (88.0%)	454 (89.0%)	147 (88.0%)		
NA	25 (3.7%)	16 (3.4%)	8 (4.0%)	20 (3.9%)	5 (3.0%)		
TOBACCO SMOKE							
Smoking status							
Current smoker	199 (29.4%)	136 (28.6%)	61 (30.5%)	143 (28.0%)	56 (33.5%)	0.11	0.0142
Former smoker	150 (22.2%)	97 (20.4%)	53 (26.5%)	104 (20.4%)	46 (27.5%)		
Never smoker	328 (48.5%)	242 (50.9%)	86 (43.0%)	263 (51.6%)	65 (38.9%)		
Smoking intensity
n cig per day—median (Q1;Q3)	15 (10;20)	15 (10;20)	20 (10;20)	15 (10;20)	20 (10;20)	0.0366	0.35
Smoking duration
Years—median (Q1;Q3)	30 (20;35)	30 (16;35)	30 (20;40)	30 (20;35)	30 (20;40)	0.15	0.63
Cumulative smoking							
Pack-years—median (Q1;Q3)	20 (10;30)	20 (8;30)	23 (10;40)	20 (10;30)	21 (10;40)	0.0128	0.25
Quitting smoke
> = 15 years	81 (54.0%)	49 (50.5%)	32 (60.4%)	57 (54.8%)	24 (52.2%)	0.30	0.86
Years—median (Q1;Q3)	15 (8;23)	15 (7;22)	15 (10;25)	15 (7;22)	15 (10;26)	0.42	0.45
AIR POLLUTION							
Residence in the Tamburi neighborhood in years median (Q1;Q3)	32 (20;50)	33 (20;50)	31 (20;50)	30 (20;50)	40 (24;50)	0.88	0.12
ASBESTOS^b^
Occupational							
*N*	111 (16.4%)	72 (15.2%)	39 (19.5%)	74 (14.5%)	37 (22.2%)	0.17	0.0296
Duration in years—median (Q1;Q3)	30 (20;35)	29 (20;35)	30 (26;35)	30 (20;35)	30 (25;35)	0.24	0.21
Familial
*N*	465 (68.7%)	319 (67.2%)	144 (72.0%)	341 (66.9%)	124 (74.3%)	0.24	0.08
Duration in years—median (Q1;Q3)	30 (20;38)	30 (20;36)	30 (19;40)	30 (20;38)	30 (18;40)	0.71	0.69
Domestic
*N*	66 (9.8%)	48 (10.1%)	18 (9.0%)	48 (9.4%)	18 (10.8%)	0.78	0.65
Duration in years—median (Q1;Q3)	45 (30;54)	43 (30;54)	50 (30;58)	40 (30;53)	50 (31;55)	0.51	0.19

Percentages could not add up to 100 due to rounding. BMI, body mass index; FEV1, forced expiratory volume in the 1st second; FVC, forced vital capacity. Missing information: *N* = 15 for smoking intensity; *N* = 16 for smoking duration; *N* = 27 for duration of familial exposure to asbestos; ^a^comparison between subsets: Fisher’s exact test and Wilcoxon test were applied for categorical and continuous variables, respectively; ^b^occupational exposures: workers at the steel plant or with asbestos; familial exposure: at least one family member working at the steel plant; domestic exposure: living in a home with asbestos-containing materials.

There were no substantial differences in terms of age, BMI and known history of comorbidities between the different subsets of patients. However, we observed a higher proportion of males among the subsets of patients underwent HRCT scan (*p* < 0.0001) or that were positive to TUS (*p* = 0.0009).

The subset of patients who underwent a HRCT scan was characterized by a prevalence of current or former smokers (*p* = 0.0142) and showed a higher proportion of subjects occupationally exposed to asbestos compared to patients who did not perform the second-level test (*p* = 0.0296). Patients positive to TUS were characterized by a higher smoking intensity (i.e., number of cigarettes per day and pack—years) compared to patients negative to TUS (*p* = 0.0366). Moreover, patients underwent HRCT scan or positive to TUS showed a moderate greater likely to participate to cancer screening programs.

We recorded no differences in terms of spirometry results between the cohort subsets (TUS negative/positive; HRCT performed/not performed). A restrictive ventilatory impairment was found in 32 patients with a negative TUS examination who did not agree to undergo a second-line HRCT scan, while 1 participant with a negative TUS examination and who did not agree to undergo HRCT scan showed an obstructive ventilatory impairment. Three subjects, of which two had a positive TUS examination and one a negative TUS examination, but who did not agree to undergo HRCT scan, had a mixed restrictive-obstructive ventilator impairment. These subjects were advised to perform a further specialist evaluation to clarify their pulmonary health status.

### Overall results of TUS examinations and HRCT scans

Results from TUS examinations and HRCT scans are presented in Table 2 (and online Supplementary Table S1).

**Table 2:** Results of transthoracic ultrasound examination (TUS) examinations and high resolution computed tomography examination (HRCT) scans.

	TUS results				HRCT results						*N* (%)
	Pleural line’s Thickening	Hypoechoic striae	Hypoechoic subpleural nodules	Pleural effusions	Circumscribed pleural thickening	Pleural plaques	Interstitial Abnormalities	Pulmonary nodules	Subpleural nodules	Pleural effusions	
(A) TUS positive and no HRCT scans	**66 (63.5%)**	**22 (21.2%)**	**38 (36.5%)**	**7 (6.7%)**	**-**	**-**	**-**	**-**	**-**	**-**	**104**
(B) TUS positive and HRCT scans	**63 (65.6%)**	**27 (28.1%)**	**51 (53.1%)**	**6 (6.3%)**	**22 (22.9%)**	**1 (1.0%)**	**34 (35.4%)**	**66 (66.8%)**	**43 (44.8%)**	**3 (3.1%)**	**96**
TUS true positive	52 (64.2%)	25 (30.9%)	46 (56.8%)	6 (7.4%)	22 (27.2%)	1 (1.2%)	34 (42.0%)	66 (81.5%)	43 (53.1%)	3 (3.7%)	81 (84.4%)
TUS false positive	11 (73.3%)	2 (13.3%)	5 (33.3%)	0	0	0	0	0	0	0	15 (15.6%)
(C) TUS negative and HRCT scans	0	0	0	0	**2 (2.8%)**	0	**2 (2.8%)**	**4 (5.6%)**	**3 (4.2%)**	0	**71**
TUS true negative	0	0	0	0	0	0	0	0	0	0	67 (94.4%)
TUS false negative	0	0	0	0	2 (50.0%)	0	2 (50.0%)	4 (100.0%)	3 (75.0%)	0	4 (5.6%)
*Z*-test for proportions value of *p*											
(A) vs. (B)	0.75	0.25	0.0184	0.89	-	-	-	-	-	-	-
TUS TRUE vs. FALSE POSITIVE	0.49	0.17	0.09	0.28	-	-	-	-	-	-	-

(A) TUS positive and no HRCT scans (*N* = 104); (B) TUS positive and HRCT scans (*N* = 96); (C) TUS negative and HRCT scans (*N* = 71).

A total of 675 subjects underwent TUS examination, while 167 subjects agreed to undergo both TUS examination and HRCT scan. TUS examinations detected abnormalities of the pleuro-pulmonary surface in 200 subjects. However, only 96 subjects with a positive TUS examination have agreed to undergo also a second-level HRCT scan. The remaining 104 patients with a positive TUS examination who did not undergo HRCT were advised to contact their primary clinician for further investigations.

We did not detect any major differences in the specific TUS findings among patients who underwent HRCT or not. An exception was observed for hypoechoic subpleural nodules, with a higher proportion among subjects with HRCT scan (53.1% vs. 36.5%, *p* = 0.0184). Yet, no differences were observed between TUS positive subjects with or without confirmation by the HRCT scan (TUS true positive vs. TUS false positive; Table 2).

### Tus diagnostic accuracy in the subset of patients with HRCT scans

On a total of 167 patients who agreed to undergo a second-level HRCT, TUS examination was able to actually detect presence of pleuro-pulmonary abnormalities in 81 cases (48.5%) and to exclude presence of such alterations in 67 cases (40.1%). TUS examinations failed to detect pleuro-pulmonary abnormalities in 4 patients (2.4%) with a positive HRCT scan, while 15 patients (9.0%) had a falsely positive TUS examinations despite no alterations on HRCT scan. When we compared TUS with HRCT findings, we found that TUS showed an overall diagnostic accuracy of 88.6% (82.8–93.0), with a sensitivity of 95.3% (90.8–99.8%), a specificity of 81.7% (73.3–90.1%) a positive predictive value of 84.4 (77.1–91.6) and a negative predictive value of 94.4 (89.0–99.7; Table 3).

**Table 3:** Transthoracic ultrasound examination (TUS) diagnostic accuracy in the subset with high resolution computed tomography examination (HRCT) scans (*N* = 167).

	Overall pleuro-pulmonary abnormalities	TUS Pleural line’s thickening—HRCT Interstitial abnormalities	TUS Hypoechoic striae—HRCT Interstitial abnormalities	TUS Hypoechoic subpleural nodules—HRCT Interstitial abnormalities	TUS Hypoechoic subpleural nodules—HRCT Subpleural nodules
True negative	67	91	118	101	98
False negative	4	13	22	15	18
False positive	15	40	13	30	23
True positive	81	23	14	21	28
Sensitivity	95.3 (90.8–99.8)	63.9 (48.2–79.6)	38.9 (23.0–54.8)	58.3 (42.2–74.4)	60.9 (46.8–75.0)
Specificity	81.7 (73.3–90.1)	69.5 (61.6–77.4)	90.1 (85.0–95.2)	77.1 (69.9–84.3)	81.0 (74.0–88.0)
Positive predicted value	84.4 (77.1–91.6)	36.5 (24.6–48.4)	51.9 (33.0–70.7)	41.2 (27.7–54.7)	54.9 (41.3–68.6)
Negative predicted value	94.4 (89.0–99.7)	87.5 (81.1–93.9)	84.3 (78.3–90.3)	87.1 (81.0–93.2)	84.5 (77.9–91.1)
Likelihood ratio for positive test result	5.21 (2.81–7.61)	2.09 (1.35–2.84)	3.92 (1.34–6.50)	2.55 (1.48–3.61)	3.20 (1.81–4.59)
Likelihood ratio for negative test result	0.06 (0.002–0.11)	0.52 (0.29–0.75)	0.68 (0.50–0.86)	0.54 (0.33–0.76)	0.48 (0.30–0.66)

Confidence intervals at 95% are reported in brackets.

A total of 36 patients (21.6%) presented interstitial abnormalities on HRCT scan. TUS was able to identify 34 participants presenting subpleural ILAs on HRCT scan, of whom 7 showed subpleural fibrotic ILAs (Figure 2, Clinical case 1:A–B). These subjects were referred to perform further pulmonological evaluation and, if necessary, subsequent HRCT scan to evaluate for progression. TUS was not able to detect interstitial abnormalities in 2 patients presenting non-subpleural interstitial thickening. Focusing TUS evaluation on the assessment of actual interstitial changes on HRCT, a thickened pleural line showed a sensitivity of 63.9% (48.2–79.6%) and a specificity of 69.5% (61.6–77.4%), hypoechoic striae showed a sensitivity of 38.9% (23.0–54.8%) and a specificity of 90.1% (85.0–95.2%), and subpleural nodules showed a sensitivity of 58.3% (42.2–74.4%) and a specificity of 77.1% (69.9–84.3%).

Next, we focused on the assessment of subpleural nodules. Forty six patients (27.5%) showed subpleural nodules on HRCT scan. The detection of subpleural nodules on TUS showed a sensitivity of 60.9% (46.8–75.0%) and a specificity of 81.0% (74.0–88.0%). In 24 patients (14.4%) HRCT scan revealed the presence of intraparenchymal nodules, which could not be directly imagined with TUS. Of them, 23 subjects presented a positive TUS examination for other findings. Patients with pulmonary nodules showing malignant features on HRCT scan were recommended to underwent further follow-up with low dose HRCT scan or with positron emission tomography/computed tomography (PET/CT) scan, according to the Fleischner Society’s guidelines (34). Following this evaluation, it was possible to make a diagnosis of lung cancer in 3 cases. Of them, 2 patients presented intraparenchymal nodules on HRCT scan and a TUS examination that was negative for subpleural nodules but positive for other findings (Figure 2, Clinical case 2:C–D and Clinical case 3:E–G). The remaining patient presented a big subpleural mass in the right lung that was possible to directly assess on TUS examination (Figure 2, Clinical case 4:H–J).

### Inter-observer agreement of TUS examinations

Cohen’s kappa assessed an optimal inter-readers concordance in the assessment of ultrasound findings, ranging from 0.78 for pleural effusions to 1.00 for hypoechoic subpleural nodules (online Supplementary Table S2).

## Discussion

Although the use of low-dose HRCT screening strategy in high risk smoking population was clearly demonstrated to be effective in reducing lung cancer and respiratory diseases mortality, the cost–benefit ratio of this imaging techniques is still unclear in the context of population strongly exposed to air pollution (12–17). We deemed that TUS examination is widely accessible and less expensive than other imaging methods, thus representing a valid population-wide test to reach subjects living in highly polluted areas, and that are still asymptomatic for respiratory diseases. We therefore designed a pilot study in the highly polluted district of Tamburi in Taranto (Italy), enrolling subjects with a long period of exposure (more than 10 years living in Tamburi) and between 45 and 65 years old, to limit the presence of comorbidities.

Results from this pilot study seem to support the combined employment of TUS examination and HRCT scan in a screening protocol for the early detection of pulmonary nodules and interstitial lung abnormalities in a population of asymptomatic high-risk subjects. Indeed, TUS showed an overall diagnostic accuracy of 88.6% in suggesting such pulmonary abnormalities compared to results of a second-level HRCT scan, with a sensitivity of 95.3% and a specificity of 81.7%. Presence of suspicious sonographic features on ultrasound showed a positive predictive value of 84.4% for actual pleuro-pulmonary abnormalities on HRCT; absence of suspicious sonographic features was associated to a negative predictive value of 94.4. This high predictive accuracy suggests that TUS could be effectively used as diagnostic test to identify the subset of subjects who could benefit of a second-line test with low-dose HRCT for the early diagnosis of lung cancer, as well as of interstitial lung diseases.

Tobacco smoke and environmental pollutants exposure are commonly cited as risk factors for lung cancer. Anyhow, various studies suggested that occupational and tobacco exposures are linked also with the development of ILAs (35, 36). Despite the interpretation of these incidentally detected HRCT findings is still debated, the possible progression into frank idiopathic pulmonary fibrosis (IPF) and the reported association with adverse outcomes (i.e., all-cause mortality, hospitalization, progressive functional decline, increased lung cancer risk, etc.) in some individuals, strongly suggest a potential clinical significance of ILAs. Subpleural ILAs, and in particular the subpleural fibrotic subtype, are more likely to progress and to be associated with mortality (31, 32). Recognizing patients at risk of progression may, therefore, be crucial from a therapeutic perspective, since it has been proposed the possibility of starting an early treatment with antifibrotic drugs, already employed in IPF, in such patients (37). In our pilot study, a total of 34 participants presenting suspicious sonographic findings on TUS exam showed subpleural ILAs on HRCT scan. Among them, 7 cases were classified as subpleural fibrotic ILAs. Fourteen patients revealed a reduction in both FEV_1_ and FVC values on spirometry. In the remaining 20 subjects PFTs were virtually normal. In these patients, TUS screening was, therefore, able to identify initial interstitial abnormalities before they became evident on spirometry test. All the subjects with subpleural ILAs were referred to perform further pneumological evaluations and, especially in case of fibrotic subpleural ILAs and impaired PFTs, to undergo subsequent follow-up chest HRCT scans to evaluate progression. On the contrary, TUS was not able to detect non-subpleural interstitial abnormalities in two patients, of which one showed impaired PFT results. The latter patient was clearly advised to contact their primary clinician for further investigations. However, we must underline that a restrictive ventilatory impairment needs plethysmography to be performed for confirmation (38).

The combined employment of TUS examination and HRCT scans allowed to diagnose three lung cancers. Unfortunately, in one patient the neoplasm was already in an advanced stage, leading to a rapid worsening of clinical conditions and death. On the contrary, the other two patients underwent curative resection surgery which, at the same time, allowed to make a histological diagnosis of minimally invasive adenocarcinoma. However, TUS was able to detect only the large subpleural pulmonary mass of the right upper lobe which then proved to be an advanced lung cancer. The other two pulmonary nodules, being intra-parenchymal, have not been directly imaged. The explanation relies in the physical limitations of ultrasound lung exploration. TUS is highly sensitive in detecting nodules and consolidations facing to the 70% of the echographically visible superficial pulmonary surface (if not obscured by bone structures of the thoracic cage) (19). However, the interposition of also a thin millimeter layer of air between the ultrasound beam and the lesion is sufficient to block the propagation of ultrasound, preventing the visualization of any space-occupying lesion (19, 26). Despite this limitation, the coexistence of other pulmonary alterations in high-risk individuals can increased the diagnostic sensitivity of TUS. Indeed, although the intra-parenchymal nodule resulted invisible to ultrasound, in one patient the exam was judged as “positive” because of the finding of a thickened pleural line corresponding to the presence of subpleural interstitial alterations on HRCT scan. In the other patient TUS was judged as “positive” following the finding of a hypoechoic stria which matched on the corresponding HRCT scan with the presence of a focal pleural thickening.

When the evaluation was focused on the matching between a specific pulmonary alteration (i.e., fibrotic changes or subpleural pulmonary nodules) and determinate suspicious sonographic features (i.e., pleural line’s thickening, hypoechoic striae, hypoechoic subpleural nodules) a reduction in both sensibility and specificity of TUS emerged. The absence of each sonographic finding maintained a high negative predictive value for disease, but presence of such sonographic findings had a low positive predictive value for the specific disease (i.e., lung fibrosis or subpleural pulmonary nodules). The obvious reason is that ultrasound findings are non specific. Other conditions, potentially existing in a population of subjects chronically exposed to environmental pollutants (i.e., pleural plaques, subpleural fibrosis, bronchiectasis, cysts, blebs and emphysema) may produce an irregular thickening of the hyperechoic pleural line and hypoechoic nodules and striae (19, 22). As a result, any alteration of the pleuro-pulmonary surface detected by TUS must necessarily be confirmed and characterized with a second-level HRCT scan. Furthermore, an irregular thickening of the hyperechoic pleural line, hypoechoic pleural striae and subpleural nodules are also common findings in COVID-19 pneumonia (39, 40). The final enrollment phase of our study coincided with the onset of the SARS-CoV2 pandemic in Italy. We, therefore, tried to avoid the bias related to COVID-19 by excluding from the inclusion patients who reported to have had direct contact with known COVID-19 cases or who have been affected by COVID-19 pneumonia.

Regarding the limitations of the study, we must highlight that an accurate assessment of false negatives would have more benefited from performing HRCT in almost all patients undergoing TUS examination. However, this was not possible because in the absence of positives findings by TUS or symptoms related to a suspected lung disease, there were no strictly indications for further HRCT scan and mainly because not all the patients enrolled in this study agreed to underwent the second-line HRCT assessment. As a consequence, the population for which it was effectively possible to compare TUS and HRCT results was a small fraction (25%) of the whole enrolled population. However, we did not identify major differences in the baseline characteristics between the two subsets with or without HRCT scan, thus being the group with second-line test representative of the entire population. We only noted a small prevalence of smokers and people working with asbestos among subjects who underwent the second-line test, that might have positively influenced the diagnostic performance of our screening strategy. Anyhow, as our study protocol is still ongoing, these doubts may eventually be clarified later.

Although, TUS examination is known to be an operator-dependent imaging method, we found an excellent concordance between the first and second operator in assessing the various ultrasound findings in our experience. A possible explanation is that all operators were well-trained within the same practical school of thoracic ultrasound (i.e., SIUMB Practical School of Thoracic Ultrasound in San Giovanni Rotondo). HRCT scans were interpreted by a single dedicated radiologist and this can be considered a further limitation of our study. However, characterization of pulmonary interstitial abnormalities goes beyond the scope of this research. To this regard we must underline that the diagnosis of any specific interstitial lung disease (ILD) requires consensus to be reached after discussion at a multidisciplinary conference, generally involving different clinicians and specialists, such as pulmonologists, radiologists, rheumatologists and/or pathologists (41, 42). All the subjects with subpleural ILAs were, therefore, referred to perform further specialist assessments to clarify their pulmonary health status.

## Conclusion

In conclusion, despite our results needs confirmation, contemplating the introduction of TUS as a first level examination in the screening of asymptomatic but at high-risk people may be highly advantageous in identifying subjects who need a diagnostic second-level HRCT scan. This strategy may reduce those cost-benefit limits that emerged from previous studies evaluating an annual screening with only low-dose HRCT and that currently represents the main obstacle to activate large low*-*dose HRCT screening programs in Europe.

## Data availability statement

The raw data supporting the conclusions of this article will be made available by the authors, without undue reservation.

## Ethics statement

The studies involving human participants were reviewed and approved by the Research Ethics Committee of IRCCS Fondazione Casa Sollievo della Sofferenza in San Giovanni Rotondo, Italy (Prot. N 66/CE IRCCS CSS: PLEURO-SCREENING-V1.0-15 Feb, 2017). The patients/participants provided their written informed consent to participate in this study. Written informed consent was obtained from the individual(s) for the publication of any potentially identifiable images or data included in this article.

## Author contributions

MS had the idea for and designed the study. MS, CQ, MM, BF, CB, AC, MT, EM, RT, EF, SS, MV, MG, SN, AS, AV, and MD had full access to the data and had final responsibility for the decision to submit it for publication and collected the data. MS, CQ, ED, BF, AC, and FB drafted the paper. CQ, ED, and MC did the analysis and all authors critically revised the manuscript for important intellectual content and gave final approval for the version to be published. All authors agree to be accountable for all aspects of the work in ensuring that questions related to the accuracy or integrity of any part of the work are appropriately investigated and resolved. All authors contributed to the article and approved the submitted version.

## Funding

This work was in part supported by the Italian Ministry of Health [2018-2021, GR-2016-02363975 to FB] and by the Associazione Italiana Ricerca sul Cancro [IG-22827 to FB]. The study funders had no role in the design of the study, the collection, analysis, and interpretation of the data, the writing of the manuscript, and the decision to submit the manuscript for publication.

## Conflict of interest

The authors declare that the research was conducted in the absence of any commercial or financial relationships that could be construed as a potential conflict of interest.

## Publisher’s note

All claims expressed in this article are solely those of the authors and do not necessarily represent those of their affiliated organizations, or those of the publisher, the editors and the reviewers. Any product that may be evaluated in this article, or claim that may be made by its manufacturer, is not guaranteed or endorsed by the publisher.

## Abbreviations

BaP: Benzo(a)pyrene
BDO: Benzodioxins
CO: Carbon monoxide
HRCT: High resolution computed tomography
ILAs: Interstitial lung abnormalities
IPF: Idiopathic pulmonary fibrosis
NO_2_: Nitrogen dioxide
PAH: Polycyclic aromatic hydrocarbons
PCDDs: Polychlorinated dibenzodioxins
PET/CT scan: Positron emission tomography/computed tomography scan
PFTs: Pulmonary function tests
PM_2.5_: Particulate matter 2.5
PM_10_: Particulate matter 10
SNI: Site of national interest
SO_2_: Sulfur dioxide
TGC: Time gain compensation
TUS: Thoracic ultrasound
