# Supplementary material for: Thoracic ultrasound combined with low-dose computed tomography may represent useful screening strategy in highly exposed population in the industrial city of Taranto (Italy)
Source: Front Med (Lausanne). 2023 May 16;10:1146807. doi: 10.3389/fmed.2023.1146807 (PMC10228729; doi:10.3389/fmed.2023.1146807)
Supplement: Supplementary file 1 [file Data_Sheet_1.PDF]

ONLINE SUPPLEMENTAL TABLES

Table S1. Results of TUS examinations and HRCT scans. A) TUS positive and no HRCT scans (N=104); B) TUS positive and HRCT scans (N=96); C) TUS negative and HRCT scans (N=71).

A) TUS positive and no HRCT scans

TUS results				
**Pleural line's thickening**	**Hypoechoic striae**	**Hypoechoic subpleural nodules**	**Pleural effusions**	**N (%)**
**66 (63.5%)**	**22 (21.2%)**	**38 (36.5%)**	**7 (6.7%)**	**104**
Yes	Yes	Yes	-	1 (1.0%)
Yes	Yes	-	-	4 (3.8%)
Yes	-	Yes	-	18 (17.3%)
Yes	-	-	-	43 (41.3%)
-	Yes	Yes	Yes	1 (1.0%)
-	Yes	Yes	-	1 (1.0%)
-	Yes	-	Yes	1 (1.0%)
-	Yes	-	-	14 (13.5%)
-	-	Yes	Yes	1 (1.0%)
-	-	Yes	-	16 (15.4%)
-	-	-	Yes	4 (3.8%)

	**TUS results**				**HRCT results**						
	**Pleural line's thickening**	**Hypoechoic striae**	**Hypoechoic subpleural nodules**	**Pleural effusions**	**Circumscribed pleural thickening**	**Pleural plaques**	**Interstitial abnormalities**	**Pulmonary nodules**	**Subpleural nodules**	**Pleural effusions**	**N (%)**
**B) TUS positive and HRCT scans**	**63 (65.6%)**	**27 (28.1%)**	**51 (53.1%)**	**6 (6.3%)**	**22 (22.9%)**	**1 (1.0%)**	**34 (35.4%)**	**66 (66.8%)**	**43 (44.8%)**	**3 (3.1%)**	**96**
**TUS TRUE POSITIVE**	**52 (64.2%)**	**25 (30.9%)**	**46 (56.8%)**	**6 (7.4%)**	**22 (22.9%)**	**1 (1.0%)**	**34 (35.4%)**	**66 (66.8%)**	**43 (44.8%)**	**3 (3.1%)**	**81 (84.4%)**
	Yes	Yes	Yes	-	Yes	-	Yes	Yes	-	-	1 (1.0%)
	Yes	Yes	Yes	-	Yes	-	Yes	-	-	-	1 (1.0%)
	Yes	Yes	Yes	-	-	-	Yes	Yes	Yes	-	1 (1.0%)
	Yes	Yes	Yes	-	-	-	-	Yes	Yes	-	1 (1.0%)
	Yes	Yes	Yes	-	-	-	-	Yes	-	-	2 (2.1%)
	Yes	Yes	-	Yes	Yes	-	Yes	Yes	-	-	1 (1.0%)
	Yes	Yes	-	-	Yes	-	Yes	Yes	Yes	-	1 (1.0%)
	Yes	Yes	-	-	Yes	-	Yes	Yes	-	-	1 (1.0%)
	Yes	Yes	-	-	Yes	-	Yes	-	-	-	1 (1.0%)
	Yes	Yes	-	-	-	-	Yes	Yes	-	-	2 (2.1%)
	Yes	Yes	-	-	-	-	-	Yes	Yes	-	2 (2.1%)
	Yes	Yes	-	-	-	-	-	Yes	-	-	2 (2.1%)
	Yes	-	Yes	-	Yes	-	Yes	Yes	Yes	-	2 (2.1%)
	Yes	-	Yes	-	Yes	-	Yes	Yes	-	-	1 (1.0%)
	Yes	-	Yes	-	Yes	-	Yes	-	-	-	2 (2.1%)
	Yes	-	Yes	-	Yes	-	-	Yes	Yes	-	1 (1.0%)
	Yes	-	Yes	-	-	-	Yes	Yes	Yes	-	1 (1.0%)
	Yes	-	Yes	-	-	-	Yes	-	-	-	4 (4.2%)
	Yes	-	Yes	-	-	-	-	Yes	Yes	-	7 (7.3%)
	Yes	-	Yes	-	-	-	-	Yes	-	-	1 (1.0%)
	Yes	-	-	Yes	Yes	-	Yes	Yes	Yes	Yes	1 (1.0%)
	Yes	-	-	Yes	-	-	-	Yes	-	-	1 (1.0%)
	Yes	-	-	-	-	-	Yes	Yes	-	-	1 (1.0%)
	Yes	-	-	-	-	-	Yes	-	-	-	2 (2.1%)
	Yes	-	-	-	-	-	-	Yes	Yes	-	9 (9.4%)

	Yes	-	-	-	-	-	-	Yes	-	-	3 (3.1%)
	-	Yes	Yes	-	Yes	-	Yes	Yes	Yes	-	2 (2.1%)
	-	Yes	Yes	-	-	-	-	Yes	Yes	-	1 (1.0%)
	-	Yes	-	Yes	Yes	-	Yes	Yes	-	-	1 (1.0%)
	-	Yes	-	-	Yes	-	Yes	Yes	Yes	-	1 (1.0%)
	-	Yes	-	-	-	-	Yes	-	-	-	1 (1.0%)
	-	Yes	-	-	-	-	-	Yes	-	-	3 (3.1%)
	-	-	Yes	-	Yes	Yes	-	Yes	Yes	-	1 (1.0%)
	-	-	Yes	-	Yes	-	Yes	Yes	Yes	-	1 (1.0%)
	-	-	Yes	-	Yes	-	Yes	-	-	-	2 (2.1%)
	-	-	Yes	-	Yes	-	-	Yes	Yes	-	1 (1.0%)
	-	-	Yes	-	-	-	Yes	Yes	Yes	-	1 (1.0%)
	-	-	Yes	-	-	-	Yes	-	-	-	2 (2.1%)
	-	-	Yes	-	-	-	-	Yes	Yes	-	8 (8.3%)
	-	-	Yes	-	-	-	-	Yes	-	-	2 (2.1%)
	-	-	-	Yes	-	-	-	Yes	Yes	Yes	1 (1.0%)
	-	-	-	Yes	-	-	-	Yes	-	Yes	1 (1.0%)
**TUS FALSE POSITIVE**	**11 (73.3%)**	**2 (13.3%)**	**5 (33.3%)**	-	-	-	-	-	-	-	**15 (15.6%)**
	Yes	Yes	Yes	-	-	-	-	-	-	-	1 (1.0%)
	Yes	-	Yes	-	-	-	-	-	-	-	1 (1.0%)
	Yes	-	-	-	-	-	-	-	-	-	9 (9.4%)
	-	Yes	-	-	-	-	-	-	-	-	1 (1.0%)
	-	-	Yes	-	-	-	-	-	-	-	3 (3.1%)

	**HRCT results**						**N (%)**
	**Circumscribed pleural thickening**	**Pleural plaques**	**Interstitial abnormalities**	**Pulmonary nodules**	**Subpleural nodules**	**Pleural effusions**	
**C) TUS negative and HRCT scans**	**2 (2.8%)**	**-**	**2 (2.8%)**	**4 (5.6%)**	**3 (4.2%)**	**-**	**71**
**TUS TRUE NEGATIVE**	-	-	-	-	-	-	**67 (94.4%)**
**TUS FALSE NEGATIVE**	**2 (50.0%)**	**-**	**2 (50.0%)**	**4 (100.0%)**	**3 (75.0%)**	**-**	**4 (5.6%)**
	Yes	-	Yes	Yes	Yes	-	1 (1.4%)
	Yes	-	Yes	Yes	-	-	1 (1.4%)
	-	-	-	Yes	Yes	-	2 (2.8%)

Table S2. Inter-observer agreement of TUS examinations.

		Second observer							
		Pleural line's thickening		Hypoechoic striae		Hypoechoic subpleural nodules		Pleural effusions	
		No	Yes	No	Yes	No	Yes	No	Yes
First observer	No	514	32	626	0	584	0	661	1
	Yes	3	126	1	48	0	89	4	9
	k Cohen's coefficient	0.85		0.99		1.00		0.78	
